# Six Weeks of High-Intensity Interval Training vs. Small-Sided Games: Effects on Physical Performance in Female Basketball Players

**DOI:** 10.3390/sports14050201

**Published:** 2026-05-13

**Authors:** Mima Stanković, Ilma Čaprić, Emir Biševac, Raid Mekić, Aldina Ajdinović, Zerina Salihagić, Goran Jelaska, Luka Pezelj, Igor Jelaska

**Affiliations:** 1 Faculty of Sport and Physical Education, University of Niš, 18000 Niš, Serbia; 2 Department of Biomedical Sciences, State University of Novi Pazar, 36300 Novi Pazar, Serbia; icapric@np.ac.rs (I.Č.); ebisevac@np.ac.rs (E.B.); rmekic@np.ac.rs (R.M.); aajdinovic@np.ac.rs (A.A.); zsalihagic@np.ac.rs (Z.S.); 3 Virovitica County Hospital, 33000 Virovitica, Croatia; goranjelaska@gmail.com; 4 Faculty of Maritime Studies, University of Split, 21000 Split, Croatia; luka.pezelj@pfst.hr; 5 Faculty of Kinesiology, University of Split, 21000 Split, Croatia; jelaska@kifst.hr

**Keywords:** female basketball, speed, agility, repeated sprint ability, aerobic capacity, training

## Abstract

High-intensity interval training (HIIT) and small-sided games (SSG) are popular conditioning tactics in team sports, but their relative efficiency among female basketball players is uncertain. The aim of this study was to examine and compare the effects of a six-week HIIT and SSG intervention on the physical performance of elite female basketball players. Forty-four participants (20.98 ± 4.58 years) were randomly assigned to one of three groups: HIIT (n = 14), SSG (n = 14), or control (n = 16). Pre- and post-intervention evaluations assessed sprint performance (0–10, 0–20, 0–30 m), agility (Pro-agility, Zig-zag, 9-6-3-6-9 tests), vertical jump height (CMJ, CMJA, SJ), repeated sprint ability (RSA), and aerobic capacity (VO_2_max, VIFT, MAS). HIIT and SSG significantly improved all performance measures compared to the control group (*p* < 0.001, ηp^2^ = 0.365–0.809); however, there were no significant differences between the two experimental groups. HIIT had a slightly greater effect on linear sprinting, but SSG was more effective for agility and aerobic performance. Body composition remained unchanged. These data suggest that HIIT and SSG are both effective training methods for improving speed, agility, explosive power, RSA, and aerobic capacity in female basketball players. Incorporating both strategies into an organized training program can improve sport-specific performance and overall conditioning.

## 1. Introduction

Women’s basketball is a high-intensity, intermittent sport characterized by alternating short sprints, frequent changes in direction, jumps, and contact situations, while simultaneously performing complex technical and tactical tasks [1]. Match and workload analyses in women’s basketball indicate that players perform a high number of accelerations, decelerations, jumps, and high-intensity changes in pace during games. The overall physical and mechanical demands are further increased in players occupying prominent offensive roles, such as those with pronounced shooting responsibilities, who execute a greater number of jumps, contacts, and impacts, and are exposed to higher overall mechanical loads [2]. Within this context, competitive success in women’s basketball is determined by a complex interaction between a high level of technical-tactical competence and optimally developed conditioning, functional, and motor capacities that correspond to the contemporary demands of the game [3,4,5,6,7].

Research indicates a strong multidimensional association between functional and motor performance test results and indices of competitive success in young female basketball players [7,8], and that technical skills and physical preparedness jointly explain a substantial proportion of the variance in competitive performance in women’s basketball [9,10,11]. Additionally, studies examining the structure of competitive activity and technical-tactical indicators confirm that technical-tactical mastery represents the primary component determining the effectiveness of the competitive process, while various aspects of physical and functional preparedness enable its execution under the high demands of the modern game [4,12].

Modern strength and conditioning training increasingly relies on high-intensity interval training (HIIT) and small-sided games (SSG) as specific training methods that mimic the actual demands of the game [13,14]. HIIT programs tailored to basketball have also been demonstrated to enhance female players’ physical capabilities and aerobic performance [15]. Systematic reviews and a recent meta-analysis indicate that HIIT in female basketball players and athletes from team sports leads to significant improvements in VO_2_max, speed, change-of-direction ability, explosive power, and repeated sprint ability (RSA) [16,17,18]. In young female basketball players, basketball-specific HIIT performed over a period of 5–6 weeks results in substantial improvements in aerobic performance, sprinting speed, and agility, with potential reductions in 20 m sprint times and improvements in jumping performance [15,19].

Previous research has shown that specific characteristics of SSG such as the number of players, court size, and game duration have a significant impact on physiological demands and perceived exertion in basketball [20,21]. A lower number of players and a larger playing area are associated with higher heart rate values and ratings of perceived exertion (RPE) [22]. Although SSG and high-intensity interval training (HIIT) can elicit similar heart rate responses, findings related to RPE remain inconsistent, highlighting the need for further research [13,23,24].

Previous research in the field of basketball conditioning has predominantly focused on male athletes or has examined the isolated effects of high-intensity interval training and small- sided games. It has not directly compared these two training models within identical time frames. A recurring question in coaching practice concerns whether SSG, as a more sport-specific and tactically oriented training modality, can induce comparable or even superior improvements in physical performance compared with structured HIIT. Structured HIIT allows for more precise physiological load control but is less specific to the demands of the game. Accordingly, the aim of the present study was to examine and compare the effects of a six-week SSG and HIIT program on selected indicators of physical performance in female basketball players, including speed, agility, explosive power, and aerobic capacity. It was hypothesized that both HIIT and SSG would significantly improve physical performance, with comparable overall effects, but with potential modality-specific advantages. This design enables a clearer evaluation of the effectiveness of both training models under real competitive conditions, within a time frame that is commonly applied in preparatory and competitive micro- and mesocycles.

## 2. Materials and Methods

### 2.1. Participants

Using G*Power v3.1 software (Bonn, Germany, Bonn FRG, University of Bonn), the required sample size for this research was estimated following the instructions provided in scientific literature [25]. Consequently, for a type I error α = 0.05 and a power 1-β = 0.80, a sample size of 30 to 43 was considered necessary to detect significant effects. Therefore, the sample included 44 elite female basketball players (age: 20.98 ± 4.58 years; height: 177.25 ± 7.68 cm; body weight: 70.12 ± 8.63 kg). Following baseline measurements, participants were divided into three groups randomly: HIIT (N = 14; age: 21.10 ± 4.50 years; BMI: 22.19 ± 2.84 kg/m^2^; body weight: 70.50 ± 8.40 kg; training experience: 6.20 ± 2.10 years), SSG (N = 14; age: 20.70 ± 4.80 years; BMI: 21.24 ± 2.40 kg/m^2^; body weight: 69.80 ± 8.90 kg; training experience: 6.00 ± 2.30 years), Control (C) (N = 16; age: 21.05 ± 4.60 years; BMI: 22.23 ± 2.84 kg/m^2^; body weight: 70.10 ± 8.70 kg; training experience: 6.10 ± 2.20 years). Participants were randomly assigned to either the HIIT or SSG group using a computer-generated randomization sequence, stratified by playing position to ensure balanced groups. Allocation was performed by an independent researcher using a computer-generated randomization sequence, with group assignments concealed from the researchers responsible for testing and training. The process outlined by Schulz [26] was followed for randomization.

Female basketball players over the age of fifteen who routinely train at least five times a week and compete at the top level of their individual leagues met the inclusion requirements. Anterior cruciate ligament surgery during the preceding year, lower limb injuries within the last three months, current rehabilitation, and any cardiovascular, respiratory, or other systemic disorders were among the exclusion criteria.

Players who missed more than two training sessions (n = 3) or suffered lower limb injuries during the intervention (n = 2) were excluded from the analysis.

Each subject provided written informed consent to participate after being briefed about the experimental design and testing protocols, along with their legal guardians. The University of Nis’s Human Research Ethics Committee approved the study protocol, which adhered to the principles of the Helsinki Declaration (ref no. 04-92/2, date approval: 21 January 2025). Participants and club management received a thorough explanation of the study’s methods.

### 2.2. Procedures

Over the course of two days, all participants’ physical performance was measured at baseline and after the intervention in a multidisciplinary diagnostic center on an indoor basketball court with a sprung wooden floor. To reduce the impact of circadian fluctuation, all testing sessions were conducted between 9:00 and 11:00 a.m. The initial and final measurements were conducted using the same testing sequence and protocols. When software solutions were not available, data were manually entered on pre-made data sheets. In most situations, data were automatically captured and saved by direct digital input from the measurement instruments.

All participants completed a 20 min warm-up that included jogging for six minutes, dynamic stretching for five minutes, progressive running for three minutes, change-of-direction drills for three minutes, and low-intensity plyometric jumps for three minutes. On the first day, after the warm-up, participants performed repeated sprint ability (RSA) and vertical jump tests (CMJ, CMJ with arm swing (CMJA) and squat jump (SJ). Following the same warm-up, participants completed the following additional physical performance tests on the second day: the 30 m linear sprint (with split times recorded at 10 and 20 m), the Pro-agility test, the Zig-zag test, the 9–6–3–6–9 sprint test, and the 30–15 Intermittent Fitness Test (30–15 IFT). Both baseline and post-intervention assessments used the same testing sequence and protocols.

### 2.3. Physical Performance Assessment

#### 2.3.1. Speed (Running 0–30 m)

The 10 m, 20 m, and 30 m sprint tests were used to evaluate linear sprint performance. Three pairs of photocell infrared timing gates (Microgate, Polifemo Radio Light, Bolzano, Italy) were used to assess running speed. All players were acquainted with the sprinting protocols before the test. Thirty centimeters behind the first set of timing gates was designated as the starting location. From a standing start, participants were told to sprint as far as possible across a distance of thirty meters.

Split times were recorded at 10 and 20 m, and timing began automatically when athletes crossed the first timing gate at the starting line. The time needed to traverse the first ten meters of the sprint was used to assess acceleration performance. Every participant undertook three trials, with a minimum passive recovery interval of 3 min between trials [27].

#### 2.3.2. Pro-Agility Test

The Pro-agility test was used to evaluate change-of-direction abilities. Photocell timing gates (Microgate, Polifemo Radio Light, Bolzano, Italy) were used to capture sprint timings. The participants began by standing in the middle of two cones that were ten meters apart. On the first try, participants ran five meters laterally to the right or left, then changed direction and ran ten meters to the other cone. Finally, they ran five meters back through the starting line. The opposite way was carried out on the second try. When participants crossed the starting line again, the timing was automatically halted. Every participant completed three trials, taking at least three minutes to passively recover in between. Analysis was done using the best trial. The test validity and reliability have been confirmed elsewhere [28].

#### 2.3.3. Zig-Zag Test

The Zig-zag test was used to further evaluate agility. The course required repeated acceleration and deceleration because it was divided into four 5 m sections (a total of 20 m) with cones arranged at 100° angles. At the start and finish lines were two sets of photocell timing gates (Witty, Microgate, Bolzano, Italy). Participants were told to finish the course as fast as they could, starting from a standing stance with the front foot 30 cm behind the first timing gate. Up to three trials were completed by each participant, with a three-minute passive recovery period in between. For analysis, the fastest trial was selected. The test validity and reliability have been confirmed elsewhere [29].

#### 2.3.4. 9-6-3-6-9 Sprint (180 Degree Turns)

The capacity to repeatedly shift directions by 180 degrees was evaluated using the 9–6–3–6–9 sprint test. After sprinting nine meters, touching the line, and making a 180-degree turn, the participants ran three, six, and three meters in succession before finishing with a final nine-meter sprint and a 180-degree turn at each line. Participants were told to finish the test as quickly as they could. A maximum of three trials was completed by each, with a three-minute passive recovery period in between. For analysis, the fastest trial was kept.

#### 2.3.5. Vertical Jumps (CMJ, CMJA, SJ)

The countermovement jump (CMJ), CMJ with arm swing (CMJA), and squat jump (SJ) were used to measure vertical jump height. The flight time between takeoff and landing was measured using two photoelectric cells (Optojump, Microgate, Bolzano, Italy). Participants in the CMJ jumped without moving their arms after placing their hands on their hips and flexing their knees to around a 90-degree angle. The CMJA was executed in the same way, but with a free arm swing. Participants in the SJ jumped without countermovement after holding a static semi-squat position (~90° knees, hands on hips) for three seconds. Each jump test consisted of three trials separated by one minute of passive rest, with three minutes of rest between different jump tests. Analysis was done using the best performance [30].

#### 2.3.6. Repeated Sprint Ability (RSA)

Six 40 m sprints were part of the RSA test, with 20 s of passive recovery in between. At the beginning and 20 m away were two sets of photocell timing gates (Microgate, Polifemo Radio Light, Bolzano, Italy). After starting 30 cm behind the starting line, participants ran to the 20 m mark, turned, and came back. When they crossed the starting line, the timing was stopped. Before every sprint, there was a three-second countdown. Total time (RSAtotal) and mean time (RSAavg) were used to measure RSA performance. The fatigue index (FI) was computed as follows:

FI = 100 *×* [(best time (s) − worst time (s))/best time (s)]

where FI is the fatigue index, s is time on the RSA test expressed in seconds.

#### 2.3.7. 30–15 Intermittent Fitness Test

The 30–15 IFT was used to measure anaerobic and aerobic capacity. To help with pace, cones were positioned 40 m apart, with two 3 m zones in the center. Every participant ran back and forth at rates determined by auditory beeps, increasing by 0.5 km/h every 45 s, beginning at 8 km/h for 30 s. Participants walked to the closest line while recovering. When participants failed to reach the 3 m zones at the signal three times in a row or maintain the necessary pace, the test was over. Maximum heart rate (HRmax) and maximum velocity attained (VIFT) were noted. The indirect estimate of the maximum oxygen consumption in both relative and absolute values has been calculated from the 30–15 IFT results [31].

The Borg CR-10 category-ratio scale, which ranges from “very light activity” (1) to “maximal effort” (10), was used to gauge players’ ratings of perceived exertion (RPE) [32]. Following each session of HIIT and SSG, players were to verbally indicate their RPE. Before the study started, all participants had used the CR-10 scale for four weeks to track their usual training intensities in order to ensure familiarity with the scale and enhance rating accuracy.

### 2.4. Training Interventions

During the preseason, a 6-week training intervention was carried out. Players usually engaged in four weekly training sessions (about two hours each) during this period, which included a mix of running workouts of various intensities, strength and core conditioning, technical and tactical drills unique to basketball, and match play. In addition to routine basketball practice, the SSG and HIIT interventions were used three times a week.

**Figure 1:**
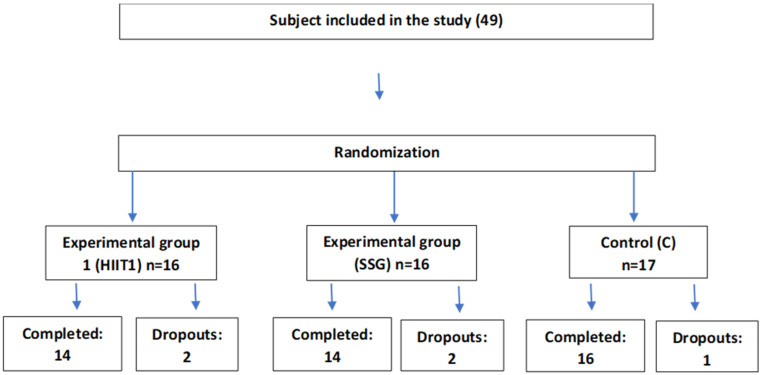
Experimental grouping diagram.

The training stimulus was gradually increased over time through adjustment of exercise duration, number of running bouts, and repetitions in both training treatments, which adhered to the progressive overload principle (Figure 1). The SSG and HIIT sessions’ duration-matched design was developed in accordance with earlier methodological guidelines [13,33]. Following a normal 15 min warm-up that included dynamic stretching, low-intensity jogging, and basketball-specific ball drills (such as dribbling, shooting, and layups), all SSG and HIIT sessions were conducted at the start of each training session. In order to properly adjust to the demands of high-intensity exercise, athletes underwent a four-week preparation training phase before the intervention.

Two-on-two games were played on half of a typical basketball court (15 × 14 m) as part of the SSG intervention. There was a two-minute passive recovery period after each bout, which lasted between two, three and four minutes. The coaching staff used strong verbal support to boost motivation, and all workouts were conducted in a competitive fashion with scores recorded. Assistant coaches with the necessary qualifications officiated each SSG. The following guidelines were used to standardize tactical and technical requirements: (a) only man-to-man defense was permitted; (b) no time-outs or free throws; (c) a 12 s shot clock; (d) play continuing upon an offensive rebound; (e) the ball had to be brought to the center circle following a successful shot or defensive rebound before starting the next attack; (f) defensive pressure was permitted right away following a change in possession or scored basket; and (g) in situations involving fouls, turnovers, or out-of-bounds situations, play was restarted by an offensive player receiving a spare ball from an assistant coach stationed at the closest sideline [34,35]. Each session’s player pairings were chosen at random, and they were changed in later sessions.

At the end of the 30–15 Intermittent Fitness Test (VIFT), the HIIT intervention comprised intermittent running bouts at 90–95% of each player’s velocity. After 15 s of running across a 20 m distance with integrated 180° direction shifts, each session concluded with 15 s of passive rest. The 180° changes in direction in the HIIT bouts were included to reflect typical basketball movement patterns. Players began the running phase in positions specific to their intended distance and completed each run concurrently at the same line. During recovery periods, players returned to their starting positions and waited for the next repetition.

### 2.5. Statistical Analysis

All data were analyzed using the statistical software package Statistica 14 Cloud Software Group, Inc.—Palo Alto, CA, USA (2023), Data Science Workbench. The normality of the data distribution was verified using the Shapiro–Wilk test, and the homogeneity of variances was confirmed using Levene’s test. Descriptive statistics were calculated for all variables and are presented as mean ± standard deviation (M ± SD) with corresponding 95% confidence intervals (95%CI). A two-way analysis of variance (two-way ANOVA) with repeated measurements was conducted to examine the main effects of the within-subjects factor Treatment (pre- vs. post-intervention) and the between-subjects factor Group (HIIT, SSG, Control), as well as the Treatment × Group interaction effect. When a significant interaction effect was detected, a Bonferroni post hoc correction was applied to identify specific differences between groups. The magnitude of the effect sizes was interpreted using partial eta squared (ηp^2^), with thresholds set as small (0.01), medium (0.06), and large (0.14). The level of statistical significance was set at *p* < 0.05 for all analyses.

## 3. Results

The descriptive statistics for all measured variables across the three groups (Control, HIIT, SSG) at baseline and following the six-week intervention are presented in Table 1. The results of the two-way ANOVA with repeated measures, including the main effects of Treatment and Group, as well as the Bonferroni post hoc comparisons, are summarized in Table 2.

Table 1 shows that both experimental groups (HIIT and SSG) demonstrated significant improvements in most physical performance measures from pre- to post-testing, with significant Group × Time interactions observed, whereas the Control group exhibited only minor changes. Specifically, the HIIT and SSG groups recorded faster sprint times across all distances (0–10 m, 0–20 m, 0–30 m), improved agility performance (Pro-agility, Zig-zag, 9-6-3-6-9), greater vertical jump heights (CMJ, CMJA, SJ), enhanced repeated sprint ability (RSA mean, RSA best, and reduced RSA fatigue index), and enhanced aerobic capacity (VO_2_max, VIFT, MAS) compared to baseline. Body composition (BMI) remained relatively stable across all groups.

Table 2 confirms a significant main effect of Treatment for virtually all performance variables (*p* < 0.001), with large effect sizes (ηp^2^ ranging from 0.365 to 0.809), indicating that the training intervention itself led to significant improvements regardless of group allocation. A significant main effect of Group was observed for the Pro-agility test (*p* = 0.045, ηp^2^ = 0.187), Zig-zag test (*p* < 0.001, ηp^2^ = 0.403), and RSA fatigue index (*p* = 0.001, ηp^2^ = 0.371), suggesting differences between the three groups in these specific measures. The Bonferroni post hoc analysis revealed that both the HIIT and SSG groups were significantly superior to the Control group in the Pro-agility test, Zig-zag test, and RSA fatigue index (HIIT-CON and SSG-CON, *p* < 0.05). No significant post hoc differences were detected between the HIIT and SSG groups for any variable, indicating that both training protocols were similarly effective in improving most aspects of physical performance in female basketball players.

## 4. Discussion

The results of this research clearly indicate that both HIIT and small-sided games (SSG) led to statistically significant improvements in most of the analyzed motor and functional variables, while changes in body composition were not significant. Overall, both training models can be considered effective, but with partially different adaptation profiles. This finding is in accordance with modern approaches to conditioning in team sports, where the complementarity of different training methods is emphasized in order to develop specific and general performances [36,37].

A significant treatment effect was found in all speed tests (0–10 m, 0–20 m, 0–30 m; *p* < 0.001), where the experimental groups made greater progress compared to the control group. HIIT showed a more pronounced effect on acceleration and linear speed, which can be explained by the specific structure of high-intensity interval work and repeated sprints. This structure stimulates neuromuscular and metabolic adaptations, including increasing the recruitment of fast motor units and improving the economy of movement at high speeds. These findings are in line with previous research, where it is stated that HIIT consistently improves sprint performance in women’s basketball [16,17], as well as that in direct comparisons with SSG, it gives greater gains in linear speed [14,37]. Additionally, research shows that specific HIIT protocols with changes in direction can have an even more pronounced effect on speed compared to linear protocols [38]. A similar pattern has been confirmed in other team sports, such as soccer [27], which further confirms the transferability of the HIIT methodology. Additionally, the post-intervention 20 m sprint values found in this study are similar to those found in elite female basketball players, where average 20 m sprint times have previously been reported to be between 3.29 and 3.52 s [39].

The most pronounced effects in this research were recorded in agility tests, especially in the Zig-zag test (ηp^2^ ≈ 0.81; *p* < 0.001), where both experimental groups were significantly more successful than the control group. SSG proved to be particularly valuable for the development of agility, which can be explained by its high specificity and the presence of open motor tasks. Namely, agility in sports does not only imply a change in direction, but also perceptual-cognitive components such as anticipation and decision-making, which are inherently present in SSG conditions. This finding is consistent with systematic reviews indicating that SSG programs consistently improve change-of-direction (COD) performance [40]. As well as research confirming the significant impact of HIIT on agility when it involves multiple changes in direction [15,38]. Additionally, SSG contributes to the improvement of technical-tactical skills, which increases its ecological validity and direct transfer to situational effectiveness in the game [14,37]. While SSG elicits more variable, cognitively demanding movement patterns that may improve reactive but less mechanically consistent change-of-direction performance, HIIT-induced improvements may be attributed to improved neuromuscular control, braking ability, and repeated high-force production during acceleration–deceleration cycles [18].

In repetitive sprint ability (RSA), statistically significant improvements were registered in all variables (*p* < 0.001), including a reduction in the fatigue index (*p* = 0.001), without significant differences between the experimental groups. These results indicate that both training models provide sufficient stimulus to improve anaerobic capacity and recovery ability between repeated efforts. These findings are consistent with research showing that HIIT significantly improves RSA and anaerobic performance [16], while SSG produces more variable but often positive effects [40]. Also, HIIT is known to increase tolerance to fatigue metabolites and the efficiency of the phosphocreatine system, while SSG contributes to specific adaptations through situationally conditioned efforts [13]. Although HIIT offers a more consistent high-intensity metabolic stimulus, SSG induces similar adaptations through variable, game-specific intermittent loading. From a physiological perspective, both HIIT and SSG likely improve repeated sprint ability through improved phosphocreatine resynthesis and glycolytic energy system efficiency [41].

Explosive strength (CMJ, CMJ arms, SJ) showed a significant treatment effect (*p* < 0.001), but no differences between groups, indicating that both training models have a similar effect on this segment. This finding is consistent with previous studies showing that HIIT may not always lead to significant changes in explosive power [15], but may contribute to its maintenance or slight improvement [37]. On the other hand, recent research indicates that certain HIIT protocols can significantly improve jump performance [19,42], which suggests that the effects depend on the training structure and the inclusion of plyometric elements. From a physiological level, increases in neuromuscular activation and stretch-shortening cycle efficiency may be linked to improvements in explosive strength. Although SSG encourages the production of reactive force through frequent, game-related jumping and landing actions, HIIT offers more structured high-force stimulus [43].

Aerobic capacity (VO_2_max, VIFT, MAS) was significantly improved in both experimental groups (*p* < 0.001), with SSG showing slightly greater improvement in VO_2_max. Additionally, the estimated VO_2_max values obtained following the intervention are within the previously reported range of 44 to 54 mL·kg^−1^·min^−1^ for female competitive basketball players [44]. This result can be explained by the higher total load and the more continuous nature of the activity within the SSG, as well as by the greater involvement of the aerobic system through prolonged periods of work of medium to high intensity. However, numerous studies confirm that HIIT also leads to a significant increase in VO_2_max, often in the range of 14 to 28% [45], as well as improvements in VIFT after 6 weeks of training [46]. According to the current findings, the SSG group’s slightly higher VO_2_max improvement could be attributed to its higher cumulative work time at moderate-to-high intensities, while HIIT probably caused stronger peak cardiovascular stress, resulting in similar overall aerobic adaptations [47].

The results of this study indicate significant positive effects of HIIT and SSG programs on physical performance in female basketball players. However, certain limitations should be considered. First, aerobic capacity (VO_2_max) was estimated using an indirect method (30–15 IFT), which may be influenced by participants’ motivation and testing conditions, potentially affecting the accuracy of the results and limiting the precision of maximal aerobic capacity estimation. Specifically, the 30–15 IFT is a field-based test utilizing performance-derived estimates instead of direct physiological VO_2_max measurement, which could lead to individual differences in effort and pacing strategies. Training adaptations and inter-individual heterogeneity in performance responses may have been further impacted by variations in recovery status, sleep quality, and energy availability. Moreover, the constancy of the administered training stimulus may have been impacted by the inability to accurately measure external and internal training loads outside of the intervention sessions. Further, as HIIT is known to cause both acute and chronic endocrine and neurotrophic responses, which may aid in performance adaptations but were not evaluated in the current study, the lack of hormonal and molecular markers should be recognized as a limitation.

Furthermore, the six-week duration of the intervention, although sufficient for initial adaptations, does not allow conclusions about long-term effects and the sustainability of the observed improvements. The absence of follow-up after the intervention represents an additional limitation. In addition, factors such as nutrition, recovery, and additional physical activities were not fully controlled and may have influenced performance outcomes. Future studies should include longer intervention periods, longitudinal monitoring, and the integration of training with other factors (e.g., nutritional interventions) in order to provide a more comprehensive understanding of the effects of different training models.

## 5. Conclusions

The results of this study demonstrate that both high-intensity interval training and small-sided games are effective training models for improving physical performance in female basketball players. Significant improvements were observed in speed, agility, repeated sprint ability, explosive power, and aerobic capacity following the six-week intervention. However, the findings also revealed partially different adaptation profiles between the two training modalities. HIIT proved to be more effective for improving linear speed and controlled high-intensity efforts, whereas SSG demonstrated greater specificity and transfer to game-related demands, particularly in agility and aerobic performance. Despite these improvements, no significant changes were observed in body composition, suggesting that longer interventions or additional factors such as nutritional control may be necessary. From a practical perspective, SSG may be given priority when combining fitness with tactical and game-specific demands, while HIIT may be emphasized during phases aimed at maximal speed and high-intensity performance.

## Figures and Tables

**Table 1:** Descriptive statistics: Mean ± standard deviation (M ± SD), 95% confidence interval (95%CI).

	Control		HIIT		SSG	
	Initial	Final	Initial	Final	Initial	Final
	M ± SD 95%CI	M ± SD 95%CI	M ± SD 95%CI	M ± SD 95%CI	M ± SD 95%CI	M ± SD 95%CI
0–10	2.07 ± 0.07 2.02–2.12	2.01 ± 0.07 1.96–2.05	2.06 ± 0.09 2.00–2.11	1.87 ± 0.20 1.73–2.00	2.09 ± 0.13 2.01–2.17	1.86 ± 0.08 1.81–1.92
0–20	3.58 ± 0.13 3.50–3.67	3.52 ± 0.16 3.41–3.62	3.55 ± 0.12 3.47–3.63	3.33 ± 0.11 3.26–3.40	3.56 ± 0.18 3.45–3.67	3.35 ± 0.22 3.19–3.50
0–30	5.04 ± 0.17 4.93–5.14	4.97 ± 0.18 4.85–5.09	5.03 ± 0.18 4.91–5.14	4.68 ± 0.15 4.58–4.77	5.05 ± 0.30 4.86–5.24	4.66 ± 0.20 4.51–4.80
PRO	5.49 ± 0.23 5.35–5.63	5.39 ± 0.23 5.24–5.53	5.47 ± 0.24 5.32–5.63	5.09 ± 0.12 5.00–5.17	5.33 ± 0.22 5.19–5.47	5.14 ± 0.14 5.04–5.24
Zig-zag	6.36 ± 0.16 6.26–6.46	6.30 ± 0.15 6.20–6.39	6.35 ± 0.16 6.25–6.46	5.66 ± 0.32 5.45–5.87	6.22 ± 0.31 6.02–6.42	5.71 ± 0.26 5.51–5.91
9-6-3-6-9	8.81 ± 0.40 8.56–9.06	8.73 ± 0.38 8.49–8.97	8.78 ± 0.40 8.53–9.04	8.31 ± 0.30 8.11–8.51	8.67 ± 0.45 8.39–8.96	8.34 ± 0.16 8.21–8.46
CMJ	23.78 ± 3.92 21.29–26.26	24.23 ± 2.64 22.55–25.90	23.78 ± 3.96 21.26–26.29	26.22 ± 2.39 24.70–27.73	22.49 ± 3.56 20.23–24.75	24.77 ± 2.87 22.94–26.59
CMJ arms	27.08 ± 4.30 23.35–29.80	28.17 ± 4.01 25.62–30.71	27.06 ± 4.31 24.32–29.80	29.43 ± 3.09 27.47–31.39	25.17 ± 4.39 22.38–27.95	28.05 ± 3.19 26.02–30.08
SJ	22.34 ± 3.27 20.26–24.42	23.43 ± 3.23 21.38–25.49	22.37 ± 3.27 29.29–24.44	24.33 ± 2.40 22.80–25.85	21.03 ± 3.55 18.78–23.29	23.71 ± 3.30 21.61–25.81
RSA mean	8.83 ± 0.38 8.59–9.07	8.63 ± 0.40 8.38–8.89	8.81 ± 0.38 8.57–9.05	8.40 ± 0.42 8.12–8.68	8.74 ± 0.43 8.46–9.01	8.27 ± 0.33 8.04–8.51
RSA best	8.30 ± 0.31 8.11–8.50	8.16 ± 0.32 7.96–8.36	8.29 ± 0.29 8.10–8.47	8.19 ± 0.35 7.96–8.42	8.28 ± 0.49 7.97–8.59	8.09 ± 0.30 7.88–8.30
RSA fatigue	12.63 ± 4.04 10.06–15.19	12.37 ± 3.90 9.89–14.84	12.64 ± 4.01 10.09–15.19	5.51 ± 1.98 4.18–6.84	10.24 ± 4.74 7.23–13.25	4.35 ± 1.64 3.18–5.53
BMI	22.23 ± 2.84 20.42–24.03	21.92 ± 2.91 20.07–23.76	22.19 ± 2.84 20.39–24.00	22.63 ± 2.87 20.81–24.46	21.24 ± 2.40 19.72–22.77	21.13 ± 2.33 19.64–22.61
30-15 VO_2_max	41.98 ± 3.13 40.00–43.97	42.50 ± 2.88 40.67–44.33	42.00 ± 3.13 40.01–43.99	43.71 ± 3.24 41.53–45.89	43.46 ± 1.38 42.58–44.34	45.95 ± 2.76 43.98–47.92
30-15 VIFT	16.15 ± 1.33 15.31–16.99	16.67 ± 1.26 15.87–17.46	16.15 ± 1.32 15.31–16.99	16.68 ± 1.23 15.86–17.51	16.41 ± 0.35 16.19–16.63	17.47 ± 0.84 16.87–18.07
30-15 MAS	4.49 ± 0.37 4.25–4.72	4.61 ± 0.37 4.38–4.84	4.49 ± 0.37 4.25–4.72	4.63 ± 0.34 4.40–4.86	4.51 ± 0.14 4.51–4.69	4.87 ± 0.22 4.71–5.03

0–10, 0–20, 0–30- running test; PRO-Pro-agility; CMJ-Countermovement jump; CMJ arms- Countermovement jump free arms; SJ-squat jump; RSA-repeated speed agility; BMI-body mass index; 30-15 MAS-maximal aerobic speed; *p* < 0.05; *p* < 0.01.

**Table 2:** Two-way repeated-measures ANOVA results for the Treatment × Group interaction, including Bonferroni post hoc comparisons. Main effect of between-subjects factor Group and within-subjects factor Treatment. F value (F), statistical significance (p), partial eta squared (ηp^2^).

	Treatment			Group			
	F	p	ηp^2^	F	p	ηp^2^	Post Hoc
0–10	36.485	<0.001	0.549	2.635	0.088	0.149	EG1-CON
0–20	28.483	<0.001	0.487	2.747	0.080	0.155	
0–30	108.424	<0.001	0.783	2.604	0.091	0.148	
PRO	59.756	<0.001	0.665	3.441	0.045	0.187	EG1-CON, EG2-CON
Zig-zag	122.62	<0.001	0.809	9.812	<0.001	0.403	EG1-CON, EG2-CON
9-6-3-6-9	73.403	<0.001	0.717	1.626	0.214	0.101	
CMJ	20.795	<0.001	0.387	0.63	0.538	0.037	
CMJ arms	36.369	<0.001	0.524	0.575	0.568	0.034	
SJ	30.433	<0.001	0.480	0.019	0.019	0.019	
RSA mean	83.743	<0.001	0.736	0.639	0.539	0.040	
RSA best	17.221	<0.001	0.365	0.041	0.960	0.003	
RSA fatigue	34.856	<0.001	0.537	8.847	0.001	0.371	EG1-CON, EG2-CON
BMI	0.001	0.973	0.000	0.681	0.513	0.040	
30-15 VO_2_max	57.477	<0.001	0.657	2.062	0.145	0.121	
30-15 VIFT	52.950	<0.001	0.638	0.770	0.472	0.049	
30-15 MAS	37.461	<0.001	0.555	1.255	0.300	0.078	

Legend: 0–10, 0–20, 0–30 running test; PRO-Pro-agility; CMJ-Countermovement jump; CMJ arms- Countermovement jump free arms; SJ-squat jump; RSA-repeated speed agility; BMI-body mass index; 30-15 MAS-maximal aerobic speed; significant difference between groups *p* < 0.05; *p* < 0.01; ηp^2^-partial eta squared.

## Data Availability

The data presented in this study are available from the corresponding author upon reasonable request. The data are not publicly available due to privacy and ethical restrictions.
